# Gut Microbiota-Related Inflammation Factors as a Potential Biomarker for Diagnosing Major Depressive Disorder

**DOI:** 10.3389/fcimb.2022.831186

**Published:** 2022-03-15

**Authors:** Shunjie Bai, Huili Bai, Detao Li, Qi Zhong, Jing Xie, Jian-jun Chen

**Affiliations:** ^1^ Department of Laboratory Medicine, The First Affiliated Hospital of Chongqing Medical University, Chongqing, China; ^2^ The Center for Clinical Molecular Medical Detection, The First Affiliated Hospital of Chongqing Medical University, Chongqing, China; ^3^ Institute of Life Sciences, Chongqing Medical University, Chongqing, China; ^4^ Chongqing Emergency Medical Center, Department of Endocrinology, The Fourth People’s Hospital of Chongqing, Central Hospital of Chongqing University, Chongqing, China

**Keywords:** major depressive disorder, gut microbiota, inflammation, Lachnospiraceae, Firmicutes

## Abstract

**Objective:**

Although many works have been done, the objectively measured diagnostic biomarkers are not available. Thus, we conducted this study to identify potential biomarkers for objectively diagnosing depression and explore the role of gut microbiota in the onset of depression.

**Methods:**

Major depressive disorder (MDD) patients (n=56) and demographic data-matched healthy controls (HCs) (n=56) were included in this study. The gut microbiota in fecal samples and inflammation-related factors in serum were measured. Both univariate and multivariate statistical analyses were performed to identify the differential gut microbiota and inflammation-related factors.

**Results:**

Finally, 46 differential operational taxonomic units (OTUs) (60.9% OTUs belonging to Firmicutes) and ten differential inflammation-related factors were identified. Correlation analysis showed that there were significant correlations between 14 differential OTUs (9 OTUs belonging to Firmicutes and 5 OTUs belonging to family Lachnospiraceae under Firmicutes) and seven differential inflammation-related factors. Meanwhile, 14 differential OTUs (9 OTUs belonging to Firmicutes and 5 OTUs belonging to family Lachnospiraceae under Firmicutes) and five differential inflammation-related factors (adiponectin, apolipoprotein A1, alpha 1-antitrypsin, neutrophilicgranulocyte count/white blood cell count and basophil count) were significantly correlated to depression severity. A panel consisting of these five differential inflammation-related factors could effectively diagnose MDD patients from HCs.

**Conclusions:**

Our results suggested that Firmicutes, especially family Lachnospiraceae, might play a role in the onset of depression *via* affecting the inflammation levels of host, and these five differential inflammation-related factors could be potential biomarkers for objectively diagnosing MDD.

## Background

Depression is a common mental disorder characterized by depressed mood, loss of appetite, and high suicide rates (Hidaka, 2012; Wachowska and Gałecki, 2021). Depression can occur at any age. It not only causes harmful effects on individuals, but also challenges the public health system with massive social and economic impacts (Rana et al., 2022). At the same time, depressive disorder has the most extensive heterogeneity of clinical diseases, which greatly increasing the difficulty of research on this type of disease. Nowadays, the identification and research of depression is not only the most complicated field of clinical scientific research, but also a major problem that needs to be solved in global life science research. Therefore, further studies are urgently needed to identify potential biomarkers for diagnosing depression and explore novel molecular mechanisms of depression.

Accumulating evidence suggests that gut microbiota is involved in the pathogenesis of depression (Bai et al., 2021a; Bai et al., 2021b; Liu L. et al., 2021). Animal studies have shown the reproducible and consistent effects of gut microbiota on mouse behavior through inflammation (Farag et al., 2020; Duan et al., 2021; Dordević et al., 2021; Zhang C. et al., 2021). Our group recently observed that CD36 deficiency could affect depressive-like behaviors possibly by modifying gut microbiota compositions in mice (Bai et al., 2021a); and gut microbiota-related metabolites held the promise as potential biomarkers for diagnosing depression (Bai et al., 2021b). Meanwhile, we found that modulation of gut microbiota compositions and the use of antibiotics could induce depressive-like behaviors (Zheng et al., 2016; Li et al., 2018). These findings demonstrated that further exploring the role of gut microbiota in depression could provide novel insights in revealing the pathogenesis of depression.

Inflammatory response is a very complex biological process, which has an important role in many diseases (Costa et al., 2021; Li et al., 2021; Lu et al., 2021; Wang J. et al., 2021). Previous studies showed that inflammatory response played a critical role in the onset of depression, and the disturbance of inflammation levels could increase the incidence of depression (Leonard, 2018; Colasanto et al., 2020). Alpha 1-antitrypsin (AAT) and apolipoprotein A1 (APOA1) were closely related with inflammatory response (Ghaiad et al., 2020; Chen et al., 2021); both were found to be significantly decreased in major depressive disorder (MDD) patients (Bai et al., 2021c). Regulating inflammatory level might be one of pathway for gut microbiota affecting host’s health. Researchers reported that the release of many inflammation factors, such as C-reactive protein (CRP), had a close relationship with gut microbiota (Pan et al., 2020; Sae-Khow et al., 2020). Meanwhile, gut microbiota-derived molecules and metabolites can result in inflammation in the central nervous system, greatly contributing to the onset of brain disorders (Barrio et al., 2021). In our previous work, we found that some inflammation-related serum metabolites were significantly correlated with gut microbiota and could be the potential biomarkers for MDD (Bai et al., 2021b). These results showed that inflammatory response might be the bridge between brain functions and gut microbiota. Therefore, we conducted this study to further explore the differences of gut microbiota at operational taxonomic units (OTUs) level and serum inflammation-related factors between MDD patients and healthy controls (HCs). In 16S rRNA gene sequencing analysis, OTUs are cluster of similar sequence variants of gene sequence, which are used to categorize bacteria based on sequence similarity. The taxonomic distributions of OTUs were used to calculate the relative abundances of gut microbiota at different levels. Our findings will improve our understanding of how gut microbiota contributed to the onset of depression.

## Methods

### MDD Patients and HCs Recruitment

The protocol of the present study was reviewed and approved by the Ethical Committee of Chongqing Medical University (No. 20200320). All MDD patients were recruited from the psychiatric center of the First Affiliated Hospital at Chongqing Medical University. The included MDD patients did not receive any antidepressive treatments in one month prior to samples collections. HCs were recruited from the medical examination center of First Affiliated Hospital at Chongqing Medical University. Finally, 56 MDD patients and 56 age, sex and body mass index (BMI)-matched HCs were included. All the included participants have provided the written informed consent. MDD diagnosis relied on a Structured Psychiatric Interview using DSM-IV-TR criteria. The detailed information was shown in **Table 1**.

**Table 1:** Demographic data of the recruited participants.

Variables	HCs	MDD patients	P-value
Number	56	56	–
Age	35.71 (15.99)	35.11 (16.79)	0.84
Sex (F/M)	36/20	38/18	0.69
BMI (kg/m^2^)	21.23 (4.27)	20.94 (2.37)	0.66
HDRS scores	0.71 (0.95)	24.86 (5.94)	<0.00001

MDD, major depressive disorder; HCs, healthy controls; Y, yes; N, no; HDRS, Hamilton Depression Rating Scale; F, female; M, male; BMI, body mass index.

### Fecal Gut Microbiota Compositions Detection

The 16S rRNA gene sequencing analysis was conducted according to the standard protocols by Majorbio Bio-Pharm Technology Co. Ltd. (Shanghai, China). Microbial DNA was extracted from fecal samples using the E.Z.N.A.^®^ soil DNA Kit (Omega Bio-tek, Norcross, GA, U.S.). Then, fecal microbiota profiling was performed by paired-end 16S rRNA gene amplicon sequencing based on the Illumina MiSeq platform (Illumina, San Diego, USA). The hypervariable regions V3-V4 of the bacterial 16S rRNA gene were used in this study. Raw 16S rRNA gene sequencing reads were demultiplexed, quality-filtered with Trimmomatic, and merged by FLASH according to the following criteria: (i) reads were truncated at any site receiving an average quality score <20 over a 50 bp sliding window. (ii) Primers were exactly matched allowing 2 nucleotides mismatching, and reads containing ambiguous bases were removed. (iii) Sequences whose overlap length longer than 10 bp were merged into one sequence. The number of allowed mismatches of barcodes was 0, and the maximum number of primer mismatches was 2. OTUs were clustered with 97% similarity cutoff using UPARSE (version 7.1 http://drive5.com/uparse/) and chimeric sequences were identified and removed using UCHIME. The taxonomy of each OTU was analyzed by Ribosomal Database Project (RDP) Classifier algorithm (http://rdp.cme.msu.edu/). The data of gut microbiota at OTU level in both groups was extracted from our previous study (Bai et al., 2021a).

### Serum Biochemical Indicator and Blood Routine Detecting

The method of serum biochemical indicators detecting was described in our previous study (Chen et al., 2021). Serum indicators of hepatic function (PALB, prealbumin; TP, total protein; ALB, albumin; GLB, globulin), lipids (TC, total cholesterol; TG, triglyceride; HDL-C, high-density lipoprotein cholesterol; LDL-C, low-density lipoprotein cholesterol; APOA1; APOB, apolipoprotein B), CRPHS (hypersensitive C-reactive protein), AAT and APN (adiponectin) were measured using commercially available enzymatic colorimetric assays and an automated analyzer system (Cobas 8000 modular device Roche Diagnostics, Switzerland). ALB was measured turbidimetrically at the main wavelength of 570 nm. TP was measured turbidimetrically at the wavelength of 546 nm. TC and TG was measured turbidimetrically at the wavelength of 505 nm. ALB was measured turbidimetrically at the wavelength of 570nm. HDL and LDL are Homogeneous enzymatic colorimetric tests, measured at the wavelength of 600 nm. APOA1, APOB, AAT and PA were measured by immunoturbidimetry at the wavelength of 340 nm. CRPHS was measured by immunoturbidimetry at the wavelength of 546 nm. APN was measured by immunoturbidimetry at the wavelength of 700 nm.

Cell counting and classification of the samples (WBC, white blood cell count; RBC, red blood cell count; PLT, platelet count; NEUT%, neutrophilicgranulocyte count/white blood cell count; NEUT, neutrophilicgranulocyte count; LYM%, lymphocyte count/white blood cell count; LYM, lymphocyte count; MONO%, monocyte count/white blood cell count; MONO, monocyte count; EO%, eosinophil count/white blood cell count; EO, eosinophil count; BASO%, basophil count/white blood cell count; BASO, basophil count) were measured using XN-9000 Hematology Analyzer system (Sysmex, Japan) through the instrument electrical impedance, flow cytometry, and nucleic acid fluorescence staining and other techniques.

### Statistical Analysis

Alpha diversity was calculated by the species richness indices (Chao) and species diversity indices (Shannon). Beta diversity was calculated by orthogonal partial least squares discriminant analysis (OPLS-DA). Linear Discriminant Analysis (LDA) Effect Size (LEfSe) was used to identify the differential OTUs responsible for the discrimination between the two groups. Random Forest was used to find out the differential inflammation-related factors responsible for the discrimination between the two groups. Pearson correlation coefficient was used to describe the correlations between differential OTUs, differential inflammation-related factors and depression severity. SPSS 19.0 and R 4.0 were used to do the statistical analyses, and P value was set to be 0.05.

## Results

### Gut Microbiota Compositions in Two Groups

**Figure 1:**
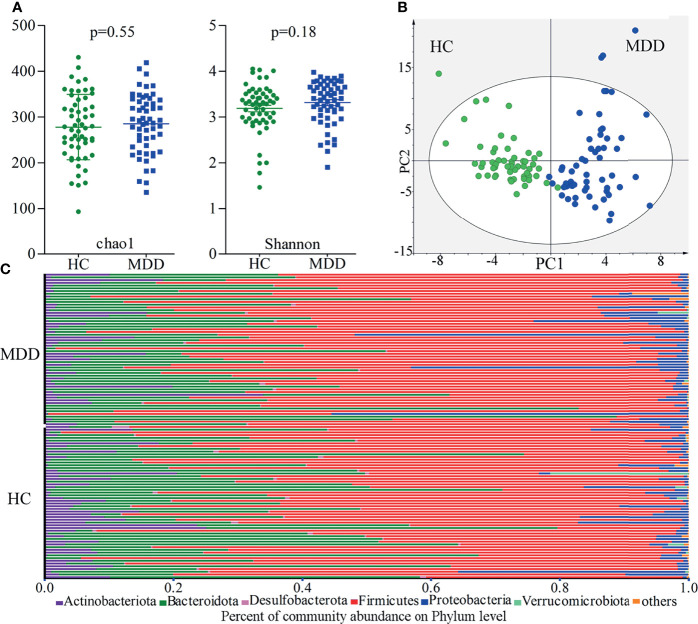
Gut microbiota compositions in MDD patients and HCs. **(A)** No significant differences on within-sample (α) phylogenetic diversity between MDD patients and HCs were identified; **(B)** β-diversity analysis showed that there were significant differences on gut microbiota compositions between the two groups; **(C)** the relative abundances of gut microbiota at phylum level in both groups. MDD, major depressive disorder; HCs, healthy controls.

There were no significant differences on within-sample (α) phylogenetic diversity between MDD patients and HCs (chao1, p=0.55; Shannon, p=0.18; **Figure 1A**). We used the beta diversity to assess whether or not there were significant differences on gut microbiota compositions at OTUs level between the two groups. The results of OPLS-DA showed that the two groups were obviously separated, suggesting the significantly divergent gut microbiota compositions at OTUs level between MDD patients and HCs (**Figure 1B**). The relative abundances of gut microbiota at phylum level in both groups were described in **Figure 1C**. The gut microbiota was mainly consisting of four phyla in both groups: Firmicutes, Bacteroidota, Actinobacteriota and Proteobacteria.

### Gut Microbiota Alterations in MDD Patients

**Figure 2:**
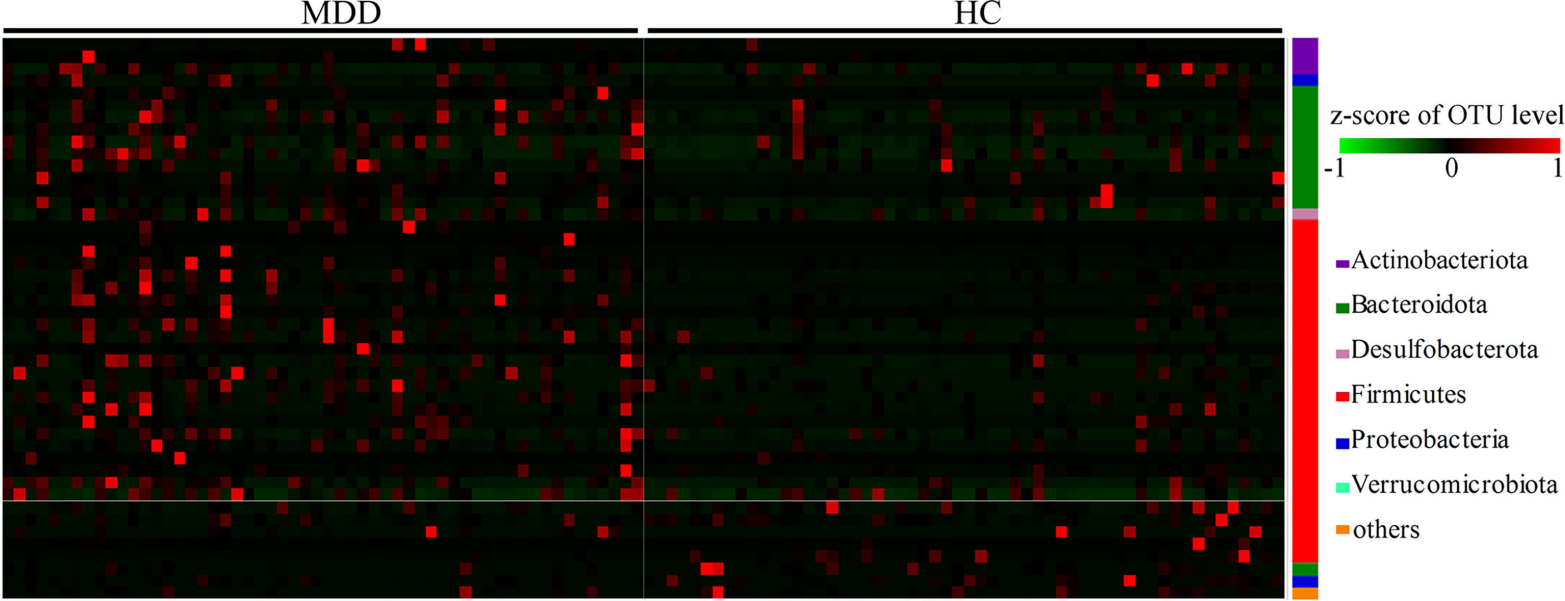
Heat-map consisting of differential OTUs between the two groups. The heat-map was built with the z-score of the relative abundance of OTUs. The redder represents the higher the abundance, and the greener represents the lower abundance. MDD, major depressive disorder; HCs, healthy controls; OTUs, operational taxonomic units.

In total, 46 OTUs responsible for the discrimination between the two groups were identified here using LEfSe analysis. The heat-map consisting of these differential OTUs showed a consistent clustering pattern in each group (**Figure 2**). As compared to HCs, MDD patients were characterized by 8 significantly decreased OTUs, along with 38 significantly increased OTUs. Among these differential OTUs, there were 28 OTUs belonging to Firmicutes (8 OTUs belonging to family Lachnospiraceae, 6 OTUs belonging to family Oscillospiraceae and 6 OTUs belonging to family Ruminococcaceae) and 11 OTUs belonging to Bacteroidota (4 OTUs belonging to family Bacteroidaceae and 3 OTUs belonging to family Rikenellaceae).

### Differential Inflammation-Related Factors in MDD Patients

**Figure 3:**
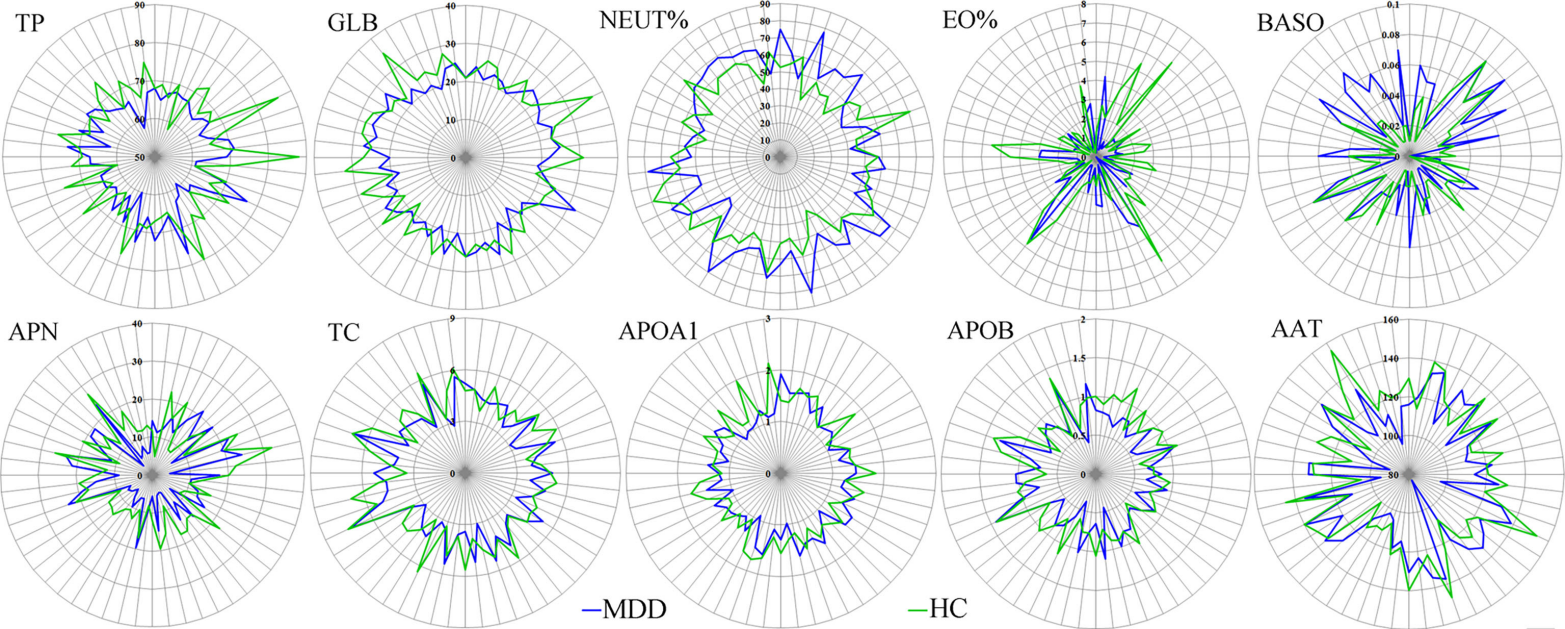
Differential inflammation-related factors in MDD patients. The number on each circle represents the levels of inflammation-related factors. The each cross-point of blue/green line and semidiameter line represents the levels of inflammation-related factors in one sample from MDD/HCs group. MDD, major depressive disorder; HCs, healthy controls.

The results of Random Forest showed that there were 15 important inflammation-related factors: TP, GLB, EO%, APN, APOB, APOA1, TC, AAT, ALB, LYM%, PALB, RBC, CRPHS, NEUT% and BASO. Univariate statistical analysis was then used to check the differential inflammation-related factors identified by multivariate statistical analysis. The results showed that 10 of 15 important inflammation-related factors remained significantly changed. The five important inflammation-related factors with p>0.05 were LYM% (p=0.109), ALB (p=0.106), PALB (p=0.129), RBC (p=0.091), and CRPHS (p=0.086). As compared to HCs, MDD patients were characterized by two significantly increased inflammation-related factors (NEUT%, p=0.033; BASO, p=0.030), along with eight significantly decreased inflammation-related factors (TP, P=0.001; GLB, p=0.002; EO%, p=0.006; APN, p=0.011; TC, p=0.023; APOA1, P=0.016; APOB, p=0.015; AAT, p=0.033). Finally, ten significantly changed inflammation-related factors were found between MDD patients and HCs (**Figure 3**).

### Correlations of Differential Variables

**Figure 4:**
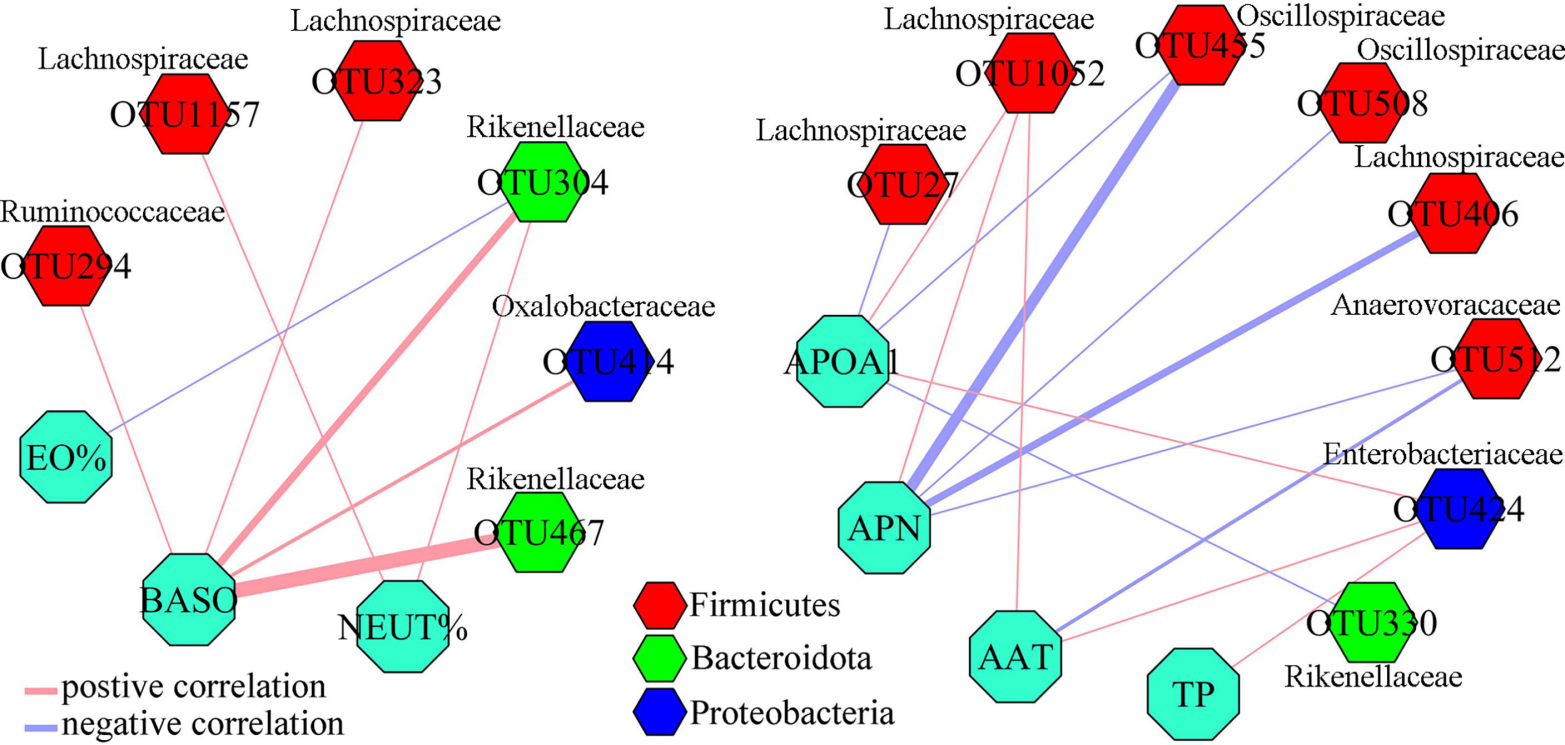
Correlations between differential OTUs and differential inflammation-related factors. Most of differential OTUs significantly related to differential inflammation-related factors belonged to Firmicutes.

To find out the potential correlations between differential gut microbiota and differential inflammation-related factors, Pearson correlation method was conducted using data from both groups. As shown in **Figure 4**, seven inflammation-related factors and 14 differential OTUs had significant correlations. The majority of differential OTUs that were significantly correlated to differential inflammation-related factors belonged to Firmicutes. Further analysis found that the OTU1052, OTU323, OTU1157, OTU406 and OTU27 belonged to family Lachnospiraceae under Firmicutes. We also found that APN was significantly correlated to five differential OTUs belonging to Firmicutes, and both APOA1 and BASO were significantly correlated to five OTUs (60% of them belonging to Firmicutes).

### HDRS-Related Differential Variables

**Figure 5:**
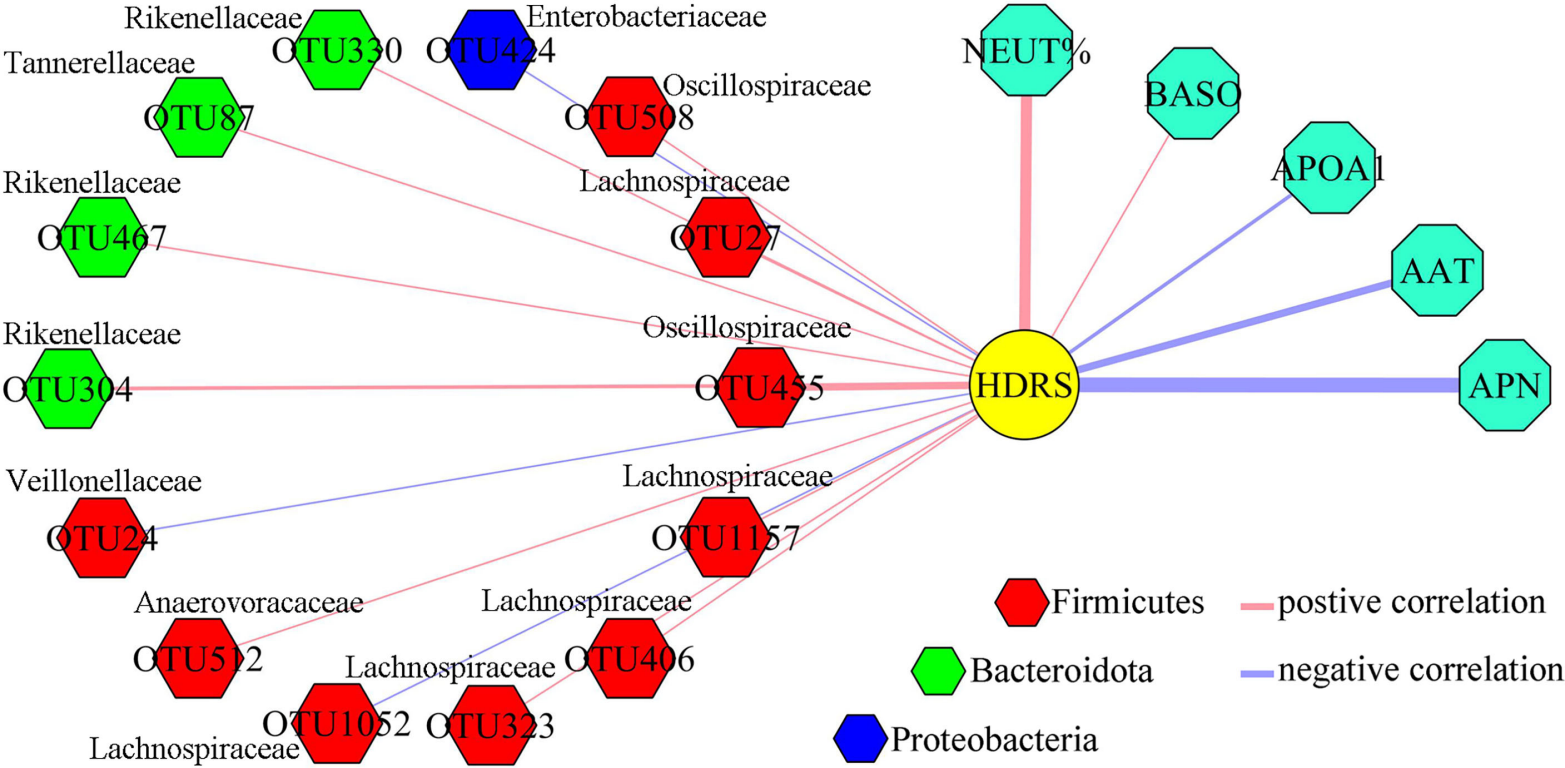
Depression severity-related differential OTUs and differential inflammation-related factors. Five depression severity-related differential inflammation factors were identified, and most of depression severity-related differential OTUs belonged to Firmicutes.

Firstly, Pearson correlation method was conducted using data from MDD patients to explore the correlations between differential inflammation-related factors and depression severity. The results showed that five differential inflammation-related factors (APN, APOA1, AAT, NEUT% and BASO) were found to be significantly correlated with HDRS (**Figure 5**). Secondly, the same method was conducted using data from MDD patients to explore the correlations between differential gut microbiota and depression severity. The results showed that 14 differential OTUs were found to be significantly correlated with HDRS (**Figure 5**). Further analysis found that 9 of 14 OUTs belonged to Firmicutes, and five OTUs (OTU323, OTU1157, OTU406, OTU27 and OTU1052) belonged to family Lachnospiraceae under Firmicutes.

### Potential Biomarkers for Diagnosing MDD

**Figure 6:**
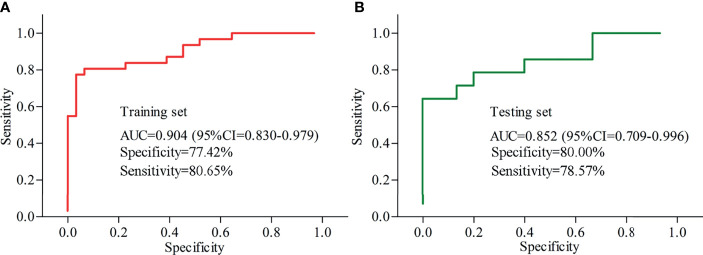
Diagnostic performances of the identified potential biomarkers. **(A)** samples in training set were used to identify the potential biomarkers, and the AUC value showed that these biomarkers could effectively diagnose MDD; **(B)** samples in testing set were used to assess the diagnostic performance of these biomarkers, and the AUC value showed that these biomarkers could effectively diagnose independent MDD patients. AUC, area under the curve; CI, confidence interval.

Logistical regression analysis was used to obtain a panel consisting of these five differential inflammation-related factors that had close relationships with depression severity and differential gut microbiota. The included MDD patients and HCs were randomly assigned into the training set and testing set on a ratio of 2:1. The samples in training set were used to identify the potential biomarkers, and the samples in testing set were used to assess the diagnostic performance of the identified potential biomarkers in predicting independent samples. In training set, the panel could effectively separate the two groups with an area under the curve (AUC) of 0.904 (95% confidence interval (CI) = 0.830-0.979) (**Figure 6A**). It is essential to use a testing set to independently validate the diagnostic performance of the panel. In testing set, we found that the panel could also effectively separate the two groups with an AUC of 0.852 (95% CI = 0.709–0.996) (**Figure 6B**).

## Discussion

In the present study, we identified 46 differential OTUs (mainly belonging to Firmicutes) and 10 differential inflammation-related factors in MDD patients. Further analysis found that seven differential inflammation-related factors were significantly correlated to 14 differential OTUs (mainly belonging to Firmicutes), and depression severity was significantly correlated to five differential inflammation-related factors (APN, APOA1, AAT, NEUT% and BASO) and 14 differential OTUs (mainly belonging to Firmicutes). In addition, five of 14 differential OTUs belonging to family Lachnospiraceae under Firmicutes were significantly correlated to these five differential inflammation-related factors and HDRS. These results indicated that Firmicutes, especially family Lachnospiraceae, might have a close relationship with depression *via* inflammation response.

Relations between gut microbiota and diseases have gained more and more attention in recent decades (Han et al., 2020; Kovács et al., 2021; Khoshkam et al., 2021; Walton et al., 2021). The significant changes of gut microbiota in MDD patients have been found in many studies (Naseribafrouei et al., 2014; Jiang et al., 2015), including ours (Zheng et al., 2016; Chen et al., 2018; Chen et al., 2020; Bai et al., 2021b). However, the specific role of gut microbiota in the onset MDD is still unknown. Previous study reported that the changes of short-chain fatty acids (SCFAs) caused by the disordered Firmicutes might account for the physiological basis of the decreased levels of inflammation in MDD patients (Huang et al., 2018). Using animal model of depression, we found that gut microbiota might contribute to the occurrence of depressive-like behaviors by affecting the levels of SCFAs and neurotransmitters (Wu et al., 2020); further investigation showed that ‘‘Firmicutes-SCFAs-GP metabolism-Tryptophan pathway” could be a potential pathway between gut microbiota and brain functions (Tian et al., 2021). Here, we observed that the majority of differential OTUs belonged to Firmicutes. Taken together, our results further demonstrated that the disturbances of Firmicutes might be a hallmark of depression.

Previous study found that the level of APOA1 was decreased in patients with post-stroke depression than in HCs (Zhan et al., 2014). Gui et al. reported that compared to HCs, MDD patients had the decreased level of AAT (Gui et al., 2018). Both APOA1 and AAT were also found to be significantly decreased in MDD patients in our previous work (Bai et al., 2021c). Liu et al. suggested that APN had an important role in depressive-like behaviors and could be a potential treatment target for MDD (Liu et al., 2012). In this study, a panel consisting of APN, APOA1, AAT, NEUT% and BASO with good diagnostic performance in diagnosing MDD was identified. Therefore, these five differential inflammation-related factors held the promise as the potential biomarkers for diagnosing MDD, and were worthy of further exploring.

As an adipocyte-derived hormone, APN plays an important role in regulating metabolism of lipids and glucose and possessing anti-inflammatory (Ouchi and Walsh, 2007; Nguyen, 2020; Zhang H. et al., 2021). Previous studies mostly focus on the relationships of APN and diabetes or cardiovascular, while the relationships between APN and depression has been increasingly attracting emphasis in recent years (Bloemer et al., 2018). Emerging evidence suggests that APN has an effect on anti-depression (Guo et al., 2017; Nicolas et al., 2018). Li et al. reported that APN could regulate the depression-related behaviors *via* acting on 5-hydroxytryptamine neurons (Li et al., 2021). Recently, researchers thought that the pathway of APN/fibroblast growth factor 9 had an important role in the development of depression (Wang X. Q. et al., 2021). Meanwhile, previous study revealed that alteration of gut microbiota could affect the expression of APN in obese mice (Yao et al., 2020). The supplementation with APN might have an impact on both the pattern and composition of the gut microbiota (Grases-Pintó et al., 2019), and APN deficiency could alter the gut microbial functions (Peng et al., 2021). Although gut microbiota has been associated with the APN, the role of APN in the gut microbiota-brain axis hypothesis of depression has not been studied to date. Here, we found that APN was significantly correlated to differential OTUs belonging to Firmicutes. Our results indicated that APN might be a critical node between gut microbiota and depression.

The gut microbiome consists of trillions of bacteria which have a critical role in host’s health (Huang et al., 2020; Khan et al., 2020; Abdullaeva et al., 2021; Piacentino et al., 2021). Firmicutes and Bacteroidetes are the two dominant bacterial phyla in human gut (Guan et al., 2021; Kameli et al., 2021). In this study, these differential OTUs mainly belonged to families under Firmicutes (Lachnospiraceae, 8 OTUs; Oscillospiraceae, 6 OTUs; Ruminococcaceae, 6 OTUs) and families under Bacteroidota (Bacteroidaceae, 4 OTUs; and Rikenellaceae, 3 OTUs). Previous studies have found that the abundances of Lachnospiraceae, Ruminococcaceae and Bacteroidaceae were changed in both depressed mice and patients (Park et al., 2020; Zhou et al., 2020; Liu X. et al., 2021). Park et al. observed that serum brain-derived neurotrophic factor was significantly correlated with Lachnospiraceae and Ruminococcaceae in individuals with depression (Park et al., 2020). Barandouzi reported that the abundances of Oscillospiraceae and Bacteroidaceae were changed in people with depression (Barandouzi et al., 2020). These results indicated that these families were worthy of further exploring.

Several limitations should be described here. Firstly, there were only 56 participants in each group, which warranted large-scale studies to validate and support our findings. Secondly, there were many overlapping symptoms between MDD and bipolar disorder (BD); thus future studies were needed to find out whether our identified potential biomarkers could separate MDD patients from BD patients. Thirdly, we did not conduct animal studies to validate the novel insights about the role of gut microbiota in the pathogenesis of depression, which was also worthy of future exploring. Fourthly, we did not know whether or not one month was enough to remove the effects of antidepressive treatments on gut microbiota; thus future studies should recruited drug-naïve MDD patients to evaluate our results. Fifthly, we only preliminarily studied the relationship between gut microbiota and inflammation factors, future studies should be further conducted to explore how the gut microbiota contributes to inflammation in host. Sixthly, although the two groups were matched in age, sex ratio and BMI, and patients with comorbidities were excluded, we still could not exclude all the confounding factors. Seventhly, other inflammatory markers, such as IL-1β and cytokines, could play an important role in the pathophysiology of MDD (Dowlati et al., 2010; Chan et al., 2019), future studies should further explore the relationships between these inflammatory markers and gut microbiota to provide novel clues in revealing the pathogenesis of depression. Finally, genetic factors were an important factor in the development of MDD; then all the participants coming from the same ethnic group might limit the general applicability of our conclusions.

## Conclusion

In conclusion, 46 differential OTUs (mainly belonging to Firmicutes) and 10 differential inflammation-related factors were identified here. Among there differential variables, 14 differential OTUs (mainly belonging to Firmicutes) and five differential inflammation-related factors (APN, APOA1, AAT, NEUT% and BASO) were found to be significantly correlated to the depression severity. Meanwhile, a panel consisting of these five inflammation-related factors could effectively diagnose MDD patients from HCs. Our results could be helpful for future exploring the role of gut microbiota in depression and identifying objectively diagnostic methods for MDD.

## Data Availability Statement

The raw data supporting the conclusions of this article will be made available by the authors, without undue reservation.

## Ethics Statement

The studies involving human participants were reviewed and approved by Ethical Committee of Chongqing Medical University. The patients/participants provided their written informed consent to participate in this study.

## Author Contributions

Study concept and design: SB and J-jC. Acquisition of data: SB, HB, and DL. Analysis and interpretation of data: All authors. Drafting of the original manuscript: SB and J-jC. Critical revision of the manuscript for important intellectual content: All authors. Statistical analysis: J-jC. Supervision: SB and J-jC. All authors read and approved the final manuscript.

## Funding

This work was supported by the Natural Science Foundation Project of China (81901398, 81701360), the Natural Science Foundation of Chongqing (cstc2021jcyj-msxmX0084, cstc2019jcyj-msxmX0025), the Science and Technology Research Program of Chongqing Municipal Education Commission (Grant No. KJQN202100420) and the Chongqing Yuzhong District Science & Technology Commission (20190115).

## Conflict of Interest

The authors declare that the research was conducted in the absence of any commercial or financial relationships that could be construed as a potential conflict of interest.

## Publisher’s Note

All claims expressed in this article are solely those of the authors and do not necessarily represent those of their affiliated organizations, or those of the publisher, the editors and the reviewers. Any product that may be evaluated in this article, or claim that may be made by its manufacturer, is not guaranteed or endorsed by the publisher.

## Abbreviations

MDD, Major depressive disorder; AAT, alpha 1-antitrypsin; CRP, C-reactive protein; HCs, healthy controls; OTUs, Operational taxonomic units; PALB, Prealbumin; TP, total protein; ALB, albumin; GLB, Globulin; TC, total Cholesterol; TG, triglyceride; HDL-C, high-density lipoprotein cholesterol; LDL-C, low-density lipoprotein cholesterol; APOA1, apolipoprotein A1; APOB, apolipoprotein B; CRPHS, hypersensitive C-reactive protein; APN, Adiponectin; WBC, White blood cell count; RBC, Red blood cell count; PLT, Platelet count; NEUT%, Neutrophilicgranulocyte count/White blood cell count; NEUT, Neutrophilicgranulocyte count; LYM%, Lymphocyte count/White blood cell count; LYM, Lymphocyte count; MONO%, Monocyte count/White blood cell count; MONO, Monocyte count; EO%, Eosinophil count/White blood cell count; EO, Eosinophil count; BASO%, Basophil count/White blood cell count; BASO, Basophil count; OPLS-DA, orthogonal partial least squares discriminant analysis; LEfSe, Linear Discriminant Analysis Effect Size; SCFAs, short-chain fatty acids.
